# Technology moral sense: Development, reliability, and validity of the TMS scale in Chinese version

**DOI:** 10.3389/fpsyg.2023.1056569

**Published:** 2023-02-02

**Authors:** Wen Wu, Xiuqing Huang, Xinyu Li

**Affiliations:** ^1^School of Marxism Studies, Zhejiang Normal University, Jinhua, China; ^2^College of Psychology, Zhejiang Normal University, Jinhua, China

**Keywords:** technology moral sense, intelligent surveillance technology, ethics, moral emotion, cognition

## Abstract

**Introduction:**

The aggregation of intelligent technologies such as big data, algorithms, and biometrics poses new moral risks to humanity and has raised awareness of technology ethics. Based on the research on moral issues in the fields of ethics and psychology, we built the concept of technology moral sense (TMS) by investigating three dimensions—technology moral consensus, cognition, and emotion.

**Methods:**

We focused on the field of intelligent surveillance technology, adopted a scale, and conducted a questionnaire survey with more than 1,000 respondents. We used exploratory and confirmatory factor analysis to test two different samples.

**Results:**

First, by combining item analysis and Cronbach’s alpha coefficient, we established that all three dimensions are reliable. Our results indicated a Cronbach’s alpha coefficient of 0.944, 0.891 and 0.938 for technology moral consensus, emotion, and cognition. Second, exploratory factor analysis verified that there were three factors, the eigenvalues were all greater than one, and the cumulative variance explanation rate was 74.953%, and the factor loading coefficient of the 18 items are greater than 0.5. Finally, we used confirmatory factor analysis to test the fit of the model. The test shows that RMSEA = 0.078, CFI and TLI are greater than 0.9, which indicating the fit was suitable and the construct validity was good.

**Discussion:**

Our findings demonstrated that the new scale is a reliable tool for assessing the technology moral sense in China. The results support the reliability and validity of the Technology Moral Sense (TMS) scale, and explain the existence of the concept of technology moral sense through three dimensions.

## Introduction

With the rapid development of technology, ethical risks caused by technology are increasing daily (Pan et al., 2018). People now find that technology is far from being as simple and beautiful as they initially thought. Technology has improved the living environment of human beings but also entails certain moral hazards (Wood, 2021). For example, intelligent surveillance technology—anchored on ubiquitous intelligence monitoring of humanity—has an increasing impact on people’s thinking patterns and behaviors (Wang and Tucker, 2021). Intelligent surveillance technology is an upgraded version of traditional surveillance technology, which integrates big data, artificial intelligence, bioengineering and other technologies to track, identify and analyze people. Intelligent surveillance technology is often used in modern life, such as intelligent monitoring, data mining, biometrics, and is widely used in public security, commerce, health care, education, entertainment and other fields. On the one hand, this technology is conducive to improving the efficiency of national governance (such as public security, epidemic prevention and control), and can also promote social and economic development; On the other hand, intelligent surveillance technology will also bring ethical challenges such as privacy disclosure, intelligent prejudice and technological hegemony, endangering human dignity and freedom. On March 20, 2022, the Chinese government issued the “Opinions on Strengthening the Ethical Governance of Science and Technology,” proposing “to formulate technology ethics norms and guidelines in the key fields such as life sciences, medicine, and artificial intelligence; improve the relevant standards of technology ethics; focus on strengthening research on technology ethics legislation” (China Government, 2022). However, research on moral sense in the technology area has not received much attention. This is because people’s moral sensitivity to technology is very low and the moral consciousness is weak (Longo et al., 2020). Moreover, people only have a vague sense of technology moral issues (Foley, 2020), and there is no suitable measuring tool to judge the moral sense of technology (Hellman, 2020). Therefore, to clarify the moral “bottom line” of key fields of technology, it is necessary to construct the concept of *technology moral sense* and develop a tool such as the TMS Scale which can provide a reliable basis for the formulation of moral norms and legislation (Martin and Shilton, 2022).

### Technology moral sense

Shaftesbury was the first scholar to propose moral sense. He believed that moral sense refers to the inherent ability of human beings to receive new emotions (Shaftesbury, 2000). Thus, moral sense encompasses the ability to generate reflective emotions, which is the unique ability to perceive moral qualities (Klein, 2001). Hutcheson further proposed a systematic theory of moral sense based on Shaftesbury. Moral sense is the premise and foundation of moral behavior, and emotional sense is the original motivation that determines the occurrence of moral behavior (Hutcheson, 2002). Hume then inherited and further developed Hutcheson’s theory of moral sense, believing that moral sense can be used as the basis for judging moral behavior (Jida, 1989).

Due to the complexity of global technology, various ethical problems arise in modern society. Further, the ethical intentions of technology use, based on the aggregate effect of technology matrices—big data mining, algorithm analysis, face recognition, positioning systems—must be examined (Verbeek, 2017). One of the strategies proposed to solve these problems is the moralization of technology (Bauman, 2000). Ihde further has pointed out that technology as an intermediary constructs people’s perception model of the world and that there are four kinds of relationships between people and technology: embodied, interpretive, other-different, and background (Ihde, 2001). Verbeek has proposed the idea of moralization of technology, arguing that technological objects can regulate people’s moral behavior and moral decision-making. Technical objects have intentionality and can become moral actors (Verbeek, 2011). Verbeek’s idea of moralizing technology provides important inspiration, showing that morality does not only occur in the human world, and technology can also cause moral issues. People who recognize the moral hazards posed in technology practice activities gradually form a moral consensus on technology, generate happy or unhappy moral emotions when applying technology, and can make moral judgments on the good and evil of technology (Moll et al., 2005; Šamánková et al., 2018). These support the theoretical connotation of moral sense in human society, so we propose the concept of technology moral sense, a lasting and stable inner experience produced by people reflecting on the moral hazards associated with technology (Waelbers, 2007).

There are various tools for measuring morality (Okan and Ekşi, 2020). In the first half of the 20th century, the famous Swiss psychologist Piaget proposed a pioneering theory of children’s moral cognitive development stages through long-term empirical research and theoretical exploration (Piaget, 1948). More than 30 years later, the American developmental psychologist Kohlberg proposed the theory of “three levels and six stages” in the theoretical construction of moral cognitive development. Some of the measurement methods he used, such as “the sentence scoring method” and “the dilemma story method,” have become classic tools of moral research (Kohlberg, 1963). Kohlberg’s student, Rest, together with his Minnesota research team, created the “four-component model,” which proposed the important factor of moral sensitivity, indicating that if people have a sense of morality, they have a sensitive understanding of the environment, sensitive awareness of moral factors and implications, and a keen awareness of the effects that actions may have on others (Rest, 1986; Rest et al., 1999). Based on the Moral Sensitivity Scale developed by Lützen et al. some scholars in various countries such as Brazil (Dalla Nora et al., 2019), South Korea (Han et al., 2010), Thailand (Tongsuebsai et al., 2015), and China (Huang et al., 2016) have measured the moral sensitivity of nurses.

The measurement methods of moral research in the field of psychology have important inspirational value and reference significance for the evaluation of technology moral sense. However, we find that there are few studies on technology moral sense, and there is no appropriate tool to measure person’s technology moral sense. Based on the connotation of moral sense, we constructed the theoretical framework for technology moral sense. Then, we designed a TMS Scale, which includes three variables: *technology moral consensus* — the basic and universal ethical principles that people believe that technology should have; *technology moral emotion* — an emotional state caused by technology; and *technology moral cognition*—the moral judgment of people about whether technology is *good* or *bad*.

### Technology moral consensus

The development of technology will have an impact on mankind all over the world, and obtaining the consensus of all mankind would be necessary. To reduce the potential moral hazards of technology, a new set of moral norms must be established based on universal values (Jobin et al., 2019). This should be congruent with the moral consensus recognized by all humanity—constant, absolute, and immutable—measured by the objects, practices, and customs that human beings strive to pursue as a community (McCauliff, 2013). Early moral sense theory paid too much attention to people’s subjective emotional judgment and ignored the objectivity and universality of the results of moral judgment (Tyler et al., 2019). The results of moral judgment should not be shifted by personal will but should truthfully reflect the original appearance of the object, be consistent, and be based on consensus. Hutcheson already proposed a certain degree of objectivity in his discussion of the object of moral sense. For example, the quality or behavior (such as loyalty and benevolence) that obtains moral approval already has moral consensus. Hume argued that since we cannot completely eradicate self-interest, it is better to transform the problem by providing an effective constraint than by the agreement concluded by all members of society, which is achieved through social interaction, a general sense of the common good manifested. Therefore, Hume overcame the subjectivity of moral sense, made it universally binding, and expanded the connotations, arguing that moral sense should include moral consensus. Similarly, when people face the moral problems brought by technology, they also need to form moral consensus. With this dimension, we can capture not only what individuals think technology is moral, but also the moral consensus that society needs (Kay and Saucier, 2020) and that can influence moral decisions (Wang et al., 2021).

### Technology moral emotion

In recent years, some research have suggested that one reminds oneself of the moral salience of a situation by producing an uncomfortable emotion, so that emotion can be used as a prerequisite for moral judgment (Decety et al., 2012). Many scholars have explored how emotions reflect person’s moral sense (Prinz, 2016). Moral emotion, especially those used to support the moral standards set by society (Haidt, 2003), come from the inherent structure of biology, past experience, and social learning (Bloom, 2013). Schoeller’s phenomenological analysis of emotions provided unique insights into the nature of moral, he pointed out that emotions have their own structure, their own cognitive dimension, and their own type of evidence (Davis and Steinbock, 2018). Past studies have shown that people have a natural or have developed an automatic ability to generate moral emotions about moral events (Greenbaum et al., 2020). Moral emotion then becomes the driving force for subsequent actions (Tangney et al., 2007). In recent years, some scholars have also put forward moral sense is a multi-dimensional structure composed of moral emotion and other variables (De Buck and Pauwels, 2019). Thomas Reid holds that external sense and moral sense have a lot in common (Broadie, 2018). Then, when people use technology, the emotion response as an external sense will arouse their technology moral sense.

### Technology moral cognition

The research on moral cognition in the field of psychology has been very mature (Baril and Wright, 2012). Cognition is not only suitable for the study of real-world problems, but also can be for in-depth understanding moral issues (Moore et al., 2019; Johnson, 2021). Inspired by philosophy, the study of moral cognition has stimulated a wave of research, such as focusing on injury and fairness, authority, and prejudice (Rottman et al., 2014). Lind (2000) argues that the description of moral behavior must include both emotional and cognitive aspects and that the same assessment tool can be used in a logically independent way to describe and measure the emotional and cognitive aspects of morality, respectively. Moreover, Lützén have proposed that there are other dimensions such as moral knowledge, moral cognition, and perception (Lützen et al., 2006). We find that the cognitive differences of technology have different effects on the moral sense of technology. For example, artificial intelligence relies heavily on human-generated data: as data enters the system, the biases of human society will be magnified. This is because intelligent algorithms may reproduce or even increase existing inequalities or discrimination (Karimi et al., 2018). Algorithms prefer aspects of phenomena and human behavior that are easy to quantify to those that are difficult or impossible to measure because some data may be easier to access and analyze than others (Ntoutsi et al., 2020). People’s moral perception is different when they have different cognitions of algorithms. Some people think algorithmic recommendation is very convenient, but some people think that algorithmic recommendation puts them in an “information cocoon” (Sima and Han, 2022).

**Figure 1:**
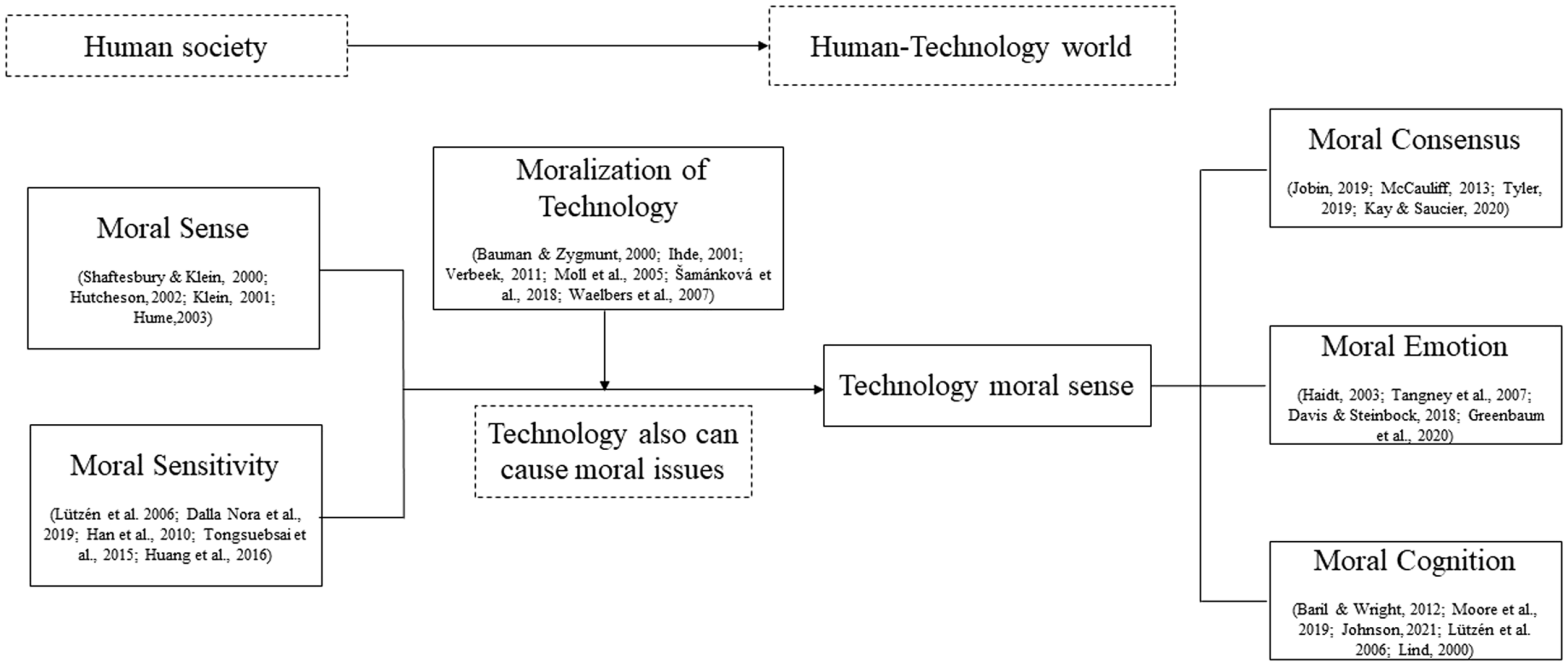
The concept evolution diagram.

Originally, moral problems only existed in human society, and people’s discussion of moral sense was limited to human beings. However, with the intervention of technology in human society, technology and people gradually interact, and technology will also cause many moral problems. Moral problems not only occur between people, but also between people and technology. However, at present, there is no concept that can describe people’s moral perception of technology. Therefore, we propose the concept: technology moral sense. As shown in Figure 1, technology moral sense is mainly intended to express people’s awareness and attitude to the moral problems caused by technology after technology enters the human society. After consulting relevant materials, we found that human society usually uses moral sense or moral sensitivity to describe people’s perception and judgment of moral issues. Therefore, we creatively put forward technology moral sense based on these two concepts, combined with the concept of moralization of technology proposed by technology philosophers. At the same time, we found that moral emotion and moral cognition are often used as variables to measure moral sense. In addition, the development of technology requires human consensus, so we added the variable of moral consensus.

In a word, this paper makes two contributions. First, we propose a new concept of technology moral sense, which is based on the three variables of technology moral consensus, technology moral emotion, and technology moral cognition. Second, we design a new tool to measure the moral sense of people when they use technology, because technology moral sense is different from the general moral sense.

## Method

In this study, we used a series of methods—including literature review, in-depth interviews, and expert judgment—to compile the items of the scale. We then conducted two questionnaire surveys with the help of a network platform to test the reliability and validity of the scale. We selected intelligent surveillance technology as an example because it is a ubiquitous and relatively common technology in people’s daily life, including intelligent monitoring, data collection, algorithm analysis, and so forth. Such techniques are familiar to people, so it is a suitable focus for public surveys. The scale was developed in two stages.

The first stage was a preliminary formulation of the items for the scale. The framework for technology moral sense was determined through a literature review. As stated in introduction section, technology moral sense is people’s reflection on the moral issues caused by technology. According to previous studies, we initially selected three dimensions of technology moral sense—consensus, emotion, and cognition.

The first dimension is *technology moral consensus*. Based on the 23 Principles of Asiloma Artificial Intelligence (Garbowski, 2018), the Principles of Technology Ethics issued by China (China Government, 2022), and the Statement on Artificial Intelligence, Robotics and “Autonomy” Systems by the European Group on Ethics in Science and New Technologies (Allan et al., 2018), we selected the principles closely related to human rights—human dignity, security, data protection, and privacy—for the items in the dimension of technology moral consensus.

The second dimension, *technology moral emotion*, focuses on understanding how people react emotionally to using technology. Emotion reminds an individual of the moral significance of a situation by bringing about discomfort, so it can be used as a prerequisite for moral judgment (Decety et al., 2012).

The third dimension—*technology moral cognition*—endeavors to understand people’s cognition of the moral hazards of technology. We adapted the Moral Foundations Questionnaire (Koleva et al., 2017) and the Moral Sensitivity Scale (Lützén et al., 1997).

We presented several hypothetical moral situations related to intelligent surveillance technology to 28 representative respondents from different fields, including government officials, employees of technology companies, homemakers, doctors, university professors, and students. Each interview lasted approximately an hour. Finally, we determined the scale items of the two dimensions of moral cognition and moral emotion. In these interviews, we found that most people could accept intelligent surveillance technology in their normal life, but given the moral hazards posed by technology, they suggested that government should strengthen the supervision of this kind of technology.

The second stage was the revision of the scale. We invited experts and scholars in the fields of psychology and ethics to evaluate the content validity of the scale. The original scale has 21 items which was then reduced to 18 items after expert evaluation. The three items (“As a technology user, I have a responsibility to know about the technology”; “I feel disrespected when personal data is being bought and sold on the market”; “It is difficult to know what technology moral issue is; my own experience is more useful than theory”) that were eliminated were considered by the experts to be of low relevance to *technology moral sense*. The experts believed that the remaining 18 items were relevant and that the items correctly reflect each dimension. The respondents rated the questions on a five-point Likert scale, ranging from strongly disagree to strongly agree. We assigned a value of 1–5 points for each item. The researchers believed the five-point Likert scale as the optimal anchor point, which was conducive to reducing the cognitive difficulty of respondents and improving the information exchange within the scale (Weijters et al., 2010; Chen et al., 2015).

We conducted two questionnaires successively. We first conducted a pre-investigation phase to initially test the applicability of the scale. To initially test the applicability of the scale, 256 questionnaires were first distributed, and 227 valid questionnaires were recovered. In the formal investigation stage, we distributed the official questionnaires nationwide using the Questionnaire Star platform. We collected 761 valid questionnaires. The recovery rate of the questionnaire is 100%. There are 59% students and 41% employers in the respondents. The vast majority (65%) was female. Academic qualification ranged from associate degree and below (12%), bachelor’s degree (73%), to master’s and above (15%). The respondents were in the nationwide including eastern region (27%), northern region (30%), southern region (33%) and western region (10%).

We used SPSS20.0 and Mplus 8.0 software for statistical analysis. At last, we adopted 18 items according to three variables. Specifically, the first variable is technology moral consensus, which refers to the universal moral principles that people think technology should follow, then we designed 5 items; The second variable is technology moral emotion, which refers to the emotional reaction of people in the process of using technology, and 6 items are designed; The third variable is technology moral cognition, which refers to people’s judgment on the moral problems existing in technology, and has designed 7 items. First, we conducted item analysis on 18 items and assigned them to high group and low group, respectively. Then, we used T test for discrimination comparison. Secondly, this study tested the reliability of three variables, using Cronbach’s alpha to measure the internal consistency of the scale, and then Exploratory Factor Analysis (EFA) was used to test the construction validity of the scale. It was mainly tested by the following indicators, such as KMO test and Bartlett’s spherical test. At the same time, it verified the number of factors. The principal component analysis method was used to extract the factors and the Kaiser standardized orthogonal rotation method is used to obtain three factors after iteration. Finally, with the new samples collected in the second time, we use the maximum likelihood estimation method to conduct Confirmatory Factor Analysis (CFA) to examine the ability of the model to fit the actual collected data, and further confirm whether the scale model is valid.

## Results

### Item analysis

Item analysis was mainly used to determine whether the items of the scale were applicable and whether the degree of distinction between the items is correctly reflected. We conducted a project analysis on 18 items, first adding up the values of the 18 items to obtain a new “total score” parameter. We sorted these in descending order of the total score and draw the first 27% and the last 27% of the sample. We assigned these as high and low groups. Samples of the same score were included within the critical value range. Next, we used a *t*-test to compare the discrimination. As shown in Table 1, the 18 items were all significant (*ps* < 0.01), which means that the 18 items were well discriminated.

**Table 1:** Project analysis results.

Item	group (mean ± standard deviation)		*t*	*p*
	high group (*N* = 71)	low group (*N* = 74)		
Q1	4.93 ± 0.258	3.59 ± 1.271	8.683	0.000^**^
Q2	4.96 ± 0.203	3.64 ± 1.351	8.163	0.000^**^
Q3	4.90 ± 0.345	3.45 ± 1.273	9.312	0.000^**^
Q4	4.63 ± 0.567	3.16 ± 1.239	9.133	0.000^**^
Q5	4.94 ± 0.232	3.68 ± 1.346	7.827	0.000^**^
Q6	4.87 ± 0.335	3.49 ± 1.274	8.881	0.000^**^
Q7	4.66 ± 0.559	3.18 ± 1.209	9.434	0.000^**^
Q8	4.03 ± 1.028	2.68 ± 0.952	8.223	0.000^**^
Q9	4.92 ± 0.280	3.38 ± 1.268	9.981	0.000^**^
Q10	4.85 ± 0.364	3.28 ± 1.153	10.899	0.000^**^
Q11	4.72 ± 0.590	3.11 ± 1.142	10.603	0.000^**^
Q12	4.80 ± 0.435	3.32 ± 1.099	10.566	0.000^**^
Q13	4.97 ± 0.167	3.70 ± 1.144	9.257	0.000^**^
Q14	4.94 ± 0.287	3.68 ± 1.183	8.783	0.000^**^
Q15	4.85 ± 0.467	3.31 ± 1.046	11.322	0.000^**^
Q16	4.82 ± 0.457	3.27 ± 1.011	11.787	0.000^**^
Q17	4.80 ± 0.467	3.39 ± 1.057	10.319	0.000^**^
Q18	4.72 ± 0.637	3.20 ± 0.993	10.892	0.000^**^

**p < 0.01.

### Reliability test

In this study, we also tested the reliability of the three dimensions of *technology moral sense* and used Cronbach’s alpha coefficient to measure the internal consistency of the scale. This is a commonly used reliability test index in scientific research. Conventionally, a Cronbach’s alpha coefficient greater than 0.85 indicates good reliability, which also means that there is internal consistency between the scores of the evaluation items, and the scale is reliable. Our results indicated a Cronbach’s alpha coefficient of 0.944, 0.891 and 0.938 for *technology moral consensus, emotion*, and *cognition*, respectively. The three indicators are all greater than 0.85, indicating that the scale has good reliability and can be used as a measuring tool for *technology moral sense*.

### Exploratory factor analysis

EFA is a method that uses factor analysis to determine dimensions and finds the multivariate internal structure of observed variables through dimensionality reduction. In advance, based on the existing theories and interviews, we assumed that technology moral sense has three dimensions: *technology moral consensus*, *emotion,* and *cognition*. We needed to clarify the existence of these factors. Additionally, the main purpose of EFA is to explore the number and the explanatory power of factors of observed variables and to maximize the information of the observed variables through constructing the factor structure (Russell, 2002). Based on the samples recovered in the pre-investigation stage, the following indicators were mainly used for testing.

First, we used the KMO test and Bartlett’s test of sphericity, to test the applicability of the scale and ensure the construct validity of the scale. We conducted KMO and Bartlett’s test of sphericity on the variables and found a KMO value of 0.947. Factor analysis is suitable when the KMO is close to 1, and there is a strong correlation between variables. A KMO of less than 0.5 indicates that the data is not suitable for factor analysis. The KMO value of this sample is very close to 1, indicating that the test is of a high standard and suitable for factor analysis. Simultaneously, the observed value of Bartlett’s test of sphericity statistic is 3999.728, and the corresponding probability value Sig is 0.000. Bartlett’s test of sphericity is used to test whether the correlation matrix is a unit matrix. If the value is large, and the corresponding probability value is significant, it indicates that the correlation coefficient matrix is significantly different from the unit matrix, and it is suitable for factor analysis.

Second, we verified the number of factors. The factors were extracted by principal component analysis, and three factors were obtained after iterating with the Kaiser normalized orthogonal rotation method. The Kaiser method is used when the standard of eigenvalue is greater than 1.0 to determine the validity of a factor. The eigenvalue represents the number of variances explained by a factor, and its value is equal to the sum of squares of factor loadings. After the applicability test of factor analysis, we tested the three factors of technology moral sense by other indicators. The eigenvalues of three factors can be seen in Table 2: technology moral consensus (factor 1) is 5.253, technology moral cognition (factor 2) is 4.455, and technology moral emotion (factor 3) is 3.784.

**Table 2:** Loading factor of technical morality factor.

Item	Factor 1	Factor 2	Factor 3	Eigenvalue	Variance explained rate (%)	Cumulative variance explained rate (%)
Q2	0.854			5.253	29.182	29.182
Q5	0.853					
Q1	0.842					
Q3	0.796					
Q4	0.721					
Q16		0.846		4.455	24.748	53.930
Q17		0.829				
Q18		0.754				
Q15		0.671				
Q14		0.646				
Q13		0.619				
Q8			0.786	3.784	21.023	74.953
Q7			0.724			
Q11			0.686			
Q9			0.603			
Q6			0.592			
Q10			0.530			
Q12			0.516			

**Figure 2:**
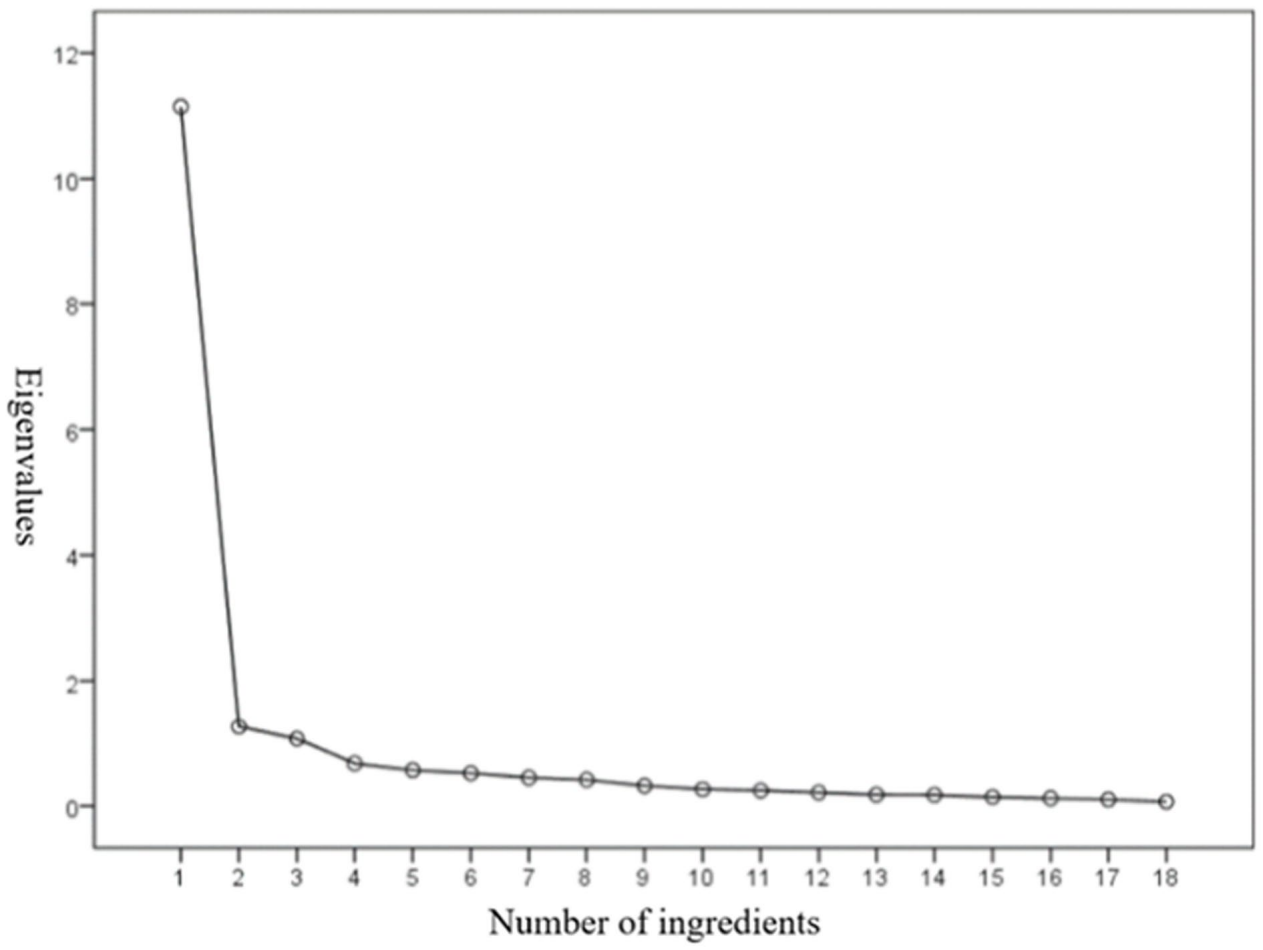
Gravel diagram.

The three eigenvalues are all greater than 1, indicating that the three factors have passed the test. The variance explanation rates are 29.182, 24.748, and 21.023%, respectively. The cumulative variance explanation rate finally reaches 74.953%, which explains nearly 75% of the feature. It means the three factors can reflect the most information about technology moral sense. Simultaneously, it can be combined with the gravel test method (Cattell, 1966). The curve of the gravel diagram (Figure 2) indicates the extracted factors at the point before the curve flattens.

The third is to check the factor loading, which is to determine whether to keep the item. Generally, there is no correlation between the factors extracted by orthogonal rotation. If the factor loading coefficient is greater than 0.5, it means that the item can better reflect the factor. A larger value indicates a closer relationship between the item and the factor. Otherwise, the item should be removed. From Table 2, it can be seen that the factor loading coefficients of the first dimension *technology moral consensus* are all greater than 0.7, indicating that the preset five items (Q2 “Technology should ensure human life first, and ensure human’s physical and mental health,” Q5 “Technology should respect human dignity,” Q1 “Technology should be aimed at enhancing human well-being.” Q3 “Technology should ensure fairness and justice to humans,” Q4 “Technology should fully obey human’s orders”) reflect the dimension *technology moral consensus* effectively.

All factor loading coefficients of the second dimension *technical moral emotion* were greater than 0.5, indicating that the preset six items (Q8 “I often feel uneasy when the access control system uses facial recognition technology,” Q7 “I often feel uncomfortable when I find out that an App will get my personal location information,” Q11 “I often feel unfair when I see a higher price for the same item on a website than the others,” Q9 “I often feel insecure when receiving some unknown calls and when I find that someone is familiar with my personal information,” Q6 “I often feel resistance when I find out that an App collects private information,” Q10 “I often receive similar information when using short video App that I will be somewhat unwilling to see”) can also effectively reflect this dimension.

The factor loading coefficients of the third dimension *technology moral cognition* were all greater than 0.6, indicating that six items (Q16 “Algorithm technology makes my life more convenient, but I think it will cause behavior control,” Q17 “Algorithm technology can help people make automated decision-making, but I think decision-making is also biased,” Q18 “I am often faced with situation which is difficult to know if the results of the application of technology are ethically correct,” Q15 “Algorithmic technology will push me similar information, but I think it will limit my freedom of information access,” Q14 “Any company can collect users’ information; I think there are privacy and security issues,” Q13 “Some software does not have my authorization before collecting my information; I think it is disrespectful to me”) reflect this dimension effectively.

Based on the above indicators, most of the items in the scale have passed the test, and the version of the scale was finally determined to be 18 items in three dimensions. However, one item in the third dimension *technology moral cognition* (Q12 “Some software collects people’s data in the background; I think it will be detrimental to people”) has a relatively low factor loading coefficient and is classified as the second dimension. To decide whether to retain this item, we conducted a CFA in subsequent new samples.

### Confirmatory factor analysis

We used CFA to test the structural validity of the evaluation model of the technology moral sense scale. By re-verifying the new samples, we examined the ability of the model to fit the other data collected and to further confirm whether the concept of the scale model was valid. The EFA showed that the test samples passed the preliminary test, indicating that the compiled technology moral sense scale met the standard and that these three dimensions could determine the variable of technology moral sense. We, therefore, continued to distribute questionnaires nationwide and collected 761 valid questionnaires. We used Mplus 8.0 software to conduct CFA on the new samples, using the maximum likelihood estimation method.

**Figure 3:**
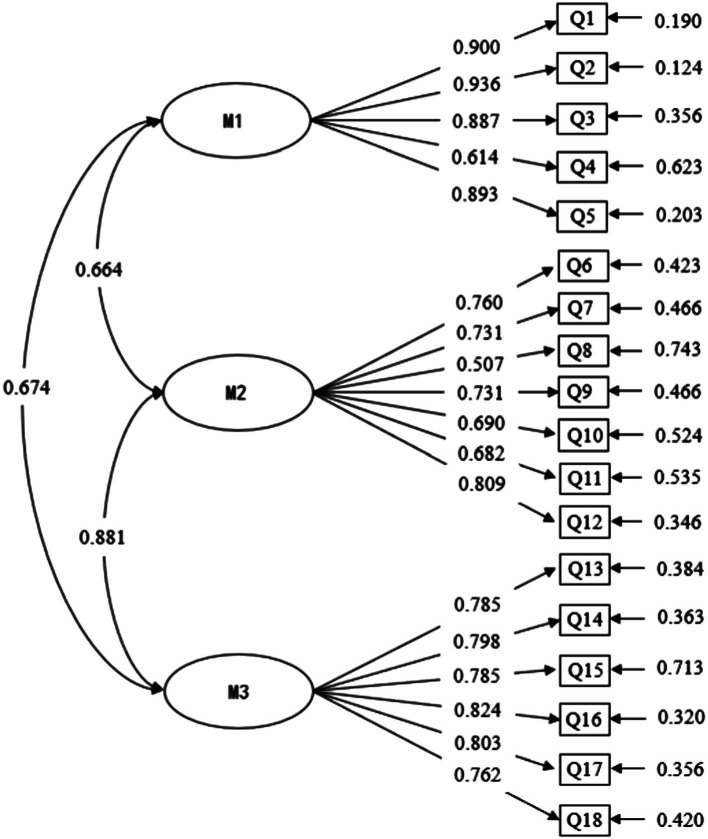
Confirmatory factor analysis model diagram.

First, we constructed a structural equation model. As latent variables in the structural equation model, technology moral consensus, technology moral emotion, and technology moral cognition were represented by M1, M2, and M3, respectively. For consistency with the EFA, the questions for each dimension were still represented by Q1–Q18. Figure 3 shows the model diagram and illustrates the factor loading coefficients of each observable variable in the model. The load coefficients of all factors were mostly above 0.7; the load coefficients of each factor were between 0.500 and 0.943; and the *p* values were all less than 0.01, indicating that the model met the standard.

Then, the program was run according to the software syntax, and the fitting index value was checked after running the program. As shown in Table 3, the first was the χ2 goodness-of-fit test. Generally, the ratio of chi-square to degrees of freedom (χ2/df) is used as its alternative test index. Through calculation, it is evident that χ2/df = 5.14, and χ2/df less than 6 indicates that the model meets the standard for a good fit. The second is to check the approximate root mean square error RMSEA, which is the best indicator to evaluate the model fitting effect. This indicator is extremely sensitive and easily interfered with by complex factors. The test shows that RMSEA = 0.078. The consensus is that RMSEA less than 0.05 indicates a very good fit, RMSEA less than 0.08 indicates a good fit and in an acceptable range, and RMSEA greater than 0.10 is considered a poor fit (Perugini and Leone, 2009). The third is to make judgments based on other fitting indices, CFI = 0.944 and TLI = 0.932. These are greater than 0.9, indicating a better degree of fit. The closer the index is to 1, the better. The fourth is to observe the standardized root mean square error SRMR. Strictly, it should be less than 0.05, but less than 0.08 is also within an acceptable range. This model SRMR = 0.056 meets the standard. The consensus is that CFA is not based on a single one. The fitting effect of the model should be investigated by combining various indicators. The indicators in this study show that the model has a good degree of fit and is suitable.

**Table 3:** Fit index of technology morality model.

*χ* ^2^	df	RMSEA	SRMR	CFI	TLI
708.893	126	0.078	0.056	0.944	0.932

## Discussion

The main purpose of this study was to design a new scale to examine a person’s technology moral sense. Since there were no tools and no prior theories had been constructed based on the relevant literature, we formally proposed the concept of *technology moral sense*. Further, we explored and designed a scale of *technology moral sense*. This study was the first to investigate the moral consensus, emotion, and cognition of technology users, and made a significant contribution to the literature because we focused on the field of intelligent surveillance technology, and conducted a questionnaire survey in China context. The TMS scale had good reliability and validity. The model can explain the existence of the concept of technology moral sense through these three dimensions. Our findings demonstrated that the new scale is a reliable tool for assessing the technology moral sense in China.

The theoretical significance is to creatively put forward the concept of technology moral sense. Due to the increasingly prominent moral issues in technology, the world needs to strengthen the ethical management of technology while developing technology. If we want to make progress in technology ethics, we need to deeply understand the perceptions and attitudes of different individuals and countries on technology moral issues. Therefore, we put forward the technology moral sense scale to evaluate whether there are differences in people’s technology moral sense among different groups and different cultural backgrounds. Of course, only a few regions in China have been investigated at present, and cross-cultural investigation can be considered in the future. Technology moral sense is a reflection of people on the moral implications of technology after ethical intentionality is evident (Hofmann, 2013). It is the ability of people to derecognize an emotion generated when using technology and reflect on its impact on themselves (Johnson and Axinn, 2013). In contrast to the previous concept of moral sense, technology moral sense needs to be discussed in a particular technology context.

The practical significance of this study lies in the model’s potential for application. Empirical research in the field of technology ethics and the measurement of scale tools can help us to timeously understand people’s attitudes and cognitions toward a certain type of technology, judge the moral risks posed by technology. This tool can also be used in other similar technology application scenarios to assess the moral hazard of technology. As a group, human beings can decide whether the technology applied now is right or wrong, and what technology should be developed cautiously. Since technology has entered the human society as a new actor, we should try our best to reach a general consensus, but the premise is that people’s perception of technology moral issues should be consistent. Only after the determination of people’s technology moral sense, can we make moral decisions or moral judgments, and finally try to establish a broad code of ethics for technology, so as to ensure the development of technology for the good.

To facilitate empirical measurement, we based the scale on the application of scenarios about intelligent surveillance technology to explore a series of hypothetical moral issues. Using the information from interviews, we designed five to six items for each dimension. We then finalized the technology moral sense scale. As shown in Table 4, the technology moral consensus includes five items from Q1 to Q5, which are used to measure people’s moral identity with the basic principles of technology ethics (such as fairness and justice, respect for human dignity); technology moral emotion includes six items from Q6 to Q12, which are used to observe people’s emotional response when they are applying technology, such as whether it makes people feel uncomfortable or resistant; technology moral cognition includes seven items from Q13 to Q18, which are used to examine people’s ability to judge the moral hazards of technology, such as privacy leaks and bias issues. From this scale, it can be found that the reliability and validity are relatively high, indicating that it has good applicability in the moral situation designed for intelligent surveillance technology. Therefore, the study extends the limitations of moral sense in human society, redefines technology moral sense, and develops a measurement tool to assess technology moral risk.

**Table 4:** Technology moral sense scale.

dimention	item	content
M1 technology moral consensus	Q1	Technology should be aimed at enhancing human well-being
	Q2	Technology should ensure human life first，and ensure human’s physical and mental health
	Q3	Technology should ensure fairness and justice to humans
	Q4	Technology should fully obey human’s orders
	Q5	Technology should respect human dignity
M2 technology moral emotion	Q6	I often feel resistance when I find out that an App collects private information
	Q7	I often feel uncomfortable when I find out that an App will get my personal location information
	Q8	I often feel uneasy when the access control system uses facial recognition technology
	Q9	I often feel insecure when receiving some unknown calls and when I find that someone is familiar with my personal information
	Q10	I often receive similar information when using short video App that I will be somewhat unwilling to see
	Q11	I often feel unfair when I see a higher price for the same item on a website than the others
M3 technology moral cognition	Q12	Some software collects people’s data in the background; I think it will be detrimental to people
	Q13	Some software does not have my authorization before collecting my information; I think it is disrespectful to me
	Q14	Any company can collect users’ information; I think there are privacy and security issues
	Q15	Algorithmic technology will push me similar information, but I think it will limit my freedom of information access
	Q16	Algorithm technology makes my life more convenient, but I think it will cause behavior control
	Q17	Algorithm technology can help people make automated decision-making, but I think decision-making is also biased
	Q18	I am often faced with situation which is difficult to know if the results of the application of technology are ethically correct

## Limitations

There are some limitations to this study. First, this study only considers people’s technology moral sense in the Chinese cultural background. Because difference cultural could affect the understanding of the concept, it needs to be further verified in other cultural backgrounds. Second, the current scale is based on technology such as big data or algorithms. More advanced technologies are likely to emerge in the future, as well as greater changes in people’s moral sense of technology. Therefore, with the advent of new technology, the scale should be constantly revised.

## Conclusion

The reliability and validity of the findings support the use of this new tool to measure technology moral sense. The three dimensions of technology moral sense are technology moral consensus, technology moral emotion, and technology moral cognition. Because the accumulation of intelligent technologies such as big data, algorithms, and biometrics pose new moral risks to humanity and has raised awareness of technology ethics, it should be clear that human society needs a unified moral consensus. We must be able to consider what technology can bring to human happiness and comfort to be able to provide technology that takes into account the need to develop ethically. This research provides an important contribution to the healthy development of technology, as few measurement tools are currently available to assess the moral hazard of technology. Moreover, the findings of this study are unique in the context of assessing people’s moral sense of technology. It would also make sense to replicate this study in other contexts. This study also provides a basis for future research on technology moral sense. In the future, we will explore the influence and function of moral intensity, personal involvement, background ambiguity, situational conditions, and other factors on technology moral sense. The relationship between technology moral sense and ethical decision-making can also be discussed. Then, we could also study whether people in different cultural backgrounds have different technology moral sense.

## Data availability statement

The original contributions presented in the study are included in the article/supplementary material, further inquiries can be directed to the corresponding authors.

## Ethics statement

Ethical review and approval was not required for the study on human participants in accordance with the local legislation and institutional requirements. Written informed consent from the [patients/participants OR patients/participants legal guardian/next of kin] was not required to participate in this study in accordance with the national legislation and the institutional requirements.

## Author contributions

WW proposes the idea and completes the manuscript. XH collects data and proofread text. XL proposes research framework. All authors contributed to the article and approved the submitted version.

## Funding

This work was funded by National Social Science Foundation of China. The project is “Research on Ethical Issues and Governance of Intelligent surveillance in Contemporary China” (Item Number: 20CZX058).

## Conflict of interest

The authors declare that the research was conducted in the absence of any commercial or financial relationships that could be construed as a potential conflict of interest.

## Publisher’s note

All claims expressed in this article are solely those of the authors and do not necessarily represent those of their affiliated organizations, or those of the publisher, the editors and the reviewers. Any product that may be evaluated in this article, or claim that may be made by its manufacturer, is not guaranteed or endorsed by the publisher.
